# The Teaching Element in Congresses

**Published:** 1893-02

**Authors:** 


### The Teaching Element in Congresses.

A new departure was inaugurated in the Dermatological Congress, recently held in Germany, by cutting down the papers to a minimum, and going a step further by suggesting that no formal business of that kind should be done at the Rome meeting next year. There is a great deal to be said for this view of the matter. If, for example, in the meetings held annually by our dental societies, the time were taken up wholly in demonstrations, and showing such cases of interest as could be collected in the neighborhood, and above all in gathering together a really well arranged museum, there is little doubt, we venture to think, that more would be learnt than by asking half-a-dozen or so persons to read papers upon subjects either too recondite for anyone to follow or too commonplace to keep men awake. Dental physiology, dental pathology and bacteriology are, as has been shown both at the last meeting of the Association, and at the Odontological Society, quite enough to draw large and interested audiences, and certainly can, when handled by good microscopists who know what they are about, be a means of conveying quite a store of useful information. The mutual intercource between men, who, living in different centres of different schools of thought, must be most beneficial, provided they are encouraged to meet for discussion and conversation. Half the advantages are lost when everyone is seated mumchance by his neighbor's side, while a lecturer raps out platitudes out of a written communication from which he never lifts his eyes. Learned papers are no doubt of great value, but they should be reserved for learned societies, where they who attend are more or less sufficiently well up in the matter to be dealt with, to understand if not discuss it. Even here much valuable time is lost, because certain men always get upon their feet whether they know anything of what they are talking about or whether they do not, and the really erudite ones either cannot speak because there is no time, or because they get in the way from sheer *ennui* of going out after the paper. Whether the first six speakers should not be either whipped up by the secretary before the meeting and duly named by the chair, may or may not be a good plan, but it certainly would enhance the value of discussions, and improve the character of our literature.—*Dental Record.*

